# Integrative analysis of blood and gut microbiota data suggests a non-alcoholic fatty liver disease (NAFLD)-related disorder in French SLA^dd^ minipigs

**DOI:** 10.1038/s41598-019-57127-x

**Published:** 2020-01-14

**Authors:** Marco Moroldo, Peris Mumbi Munyaka, Jérôme Lecardonnel, Gaëtan Lemonnier, Eric Venturi, Claire Chevaleyre, Isabelle P. Oswald, Jordi Estellé, Claire Rogel-Gaillard

**Affiliations:** 1 Université Paris Saclay, INRAE, AgroParisTech, GABI, 78350 Jouy-en-Josas, France; 2 Department of Agricultural, Food and Nutritional Science, University of Alberta, Edmonton, Canada; 3 INRAE, PAO, 37380 Nouzilly, France; 4 Université de Tours, INRAE, ISP, 37380 Nouzilly, France; 5 Université de Toulouse, INRAE, ENVT, INP-Purpan, UPS, Toxalim, 31027 Toulouse, France

**Keywords:** Molecular biology, Zoology, Gene expression, Metagenomics

## Abstract

Minipigs are a group of small-sized swine lines, which show a broad range of phenotype variation and which often tend to be obese. The SLA^dd^ (DD) minipig line was created by the NIH and selected as homozygous at the SLA locus. It was brought to France more than 30 years ago and maintained inbred ever since. In this report, we characterized the physiological status of a herd of French DD pigs by measuring intermediate phenotypes from blood and faeces and by using Large White (LW) pigs as controls. Three datasets were produced, i.e. complete blood counts (CBCs), microarray-based blood transcriptome, and faecal microbiota obtained by 16S rRNA sequencing. CBCs and expression profiles suggested a non-alcoholic fatty liver disease (NAFLD)-related pathology associated to comorbid cardiac diseases. The characterization of 16S sequencing data was less straightforward, suggesting only a potential weak link to obesity. The integration of the datasets identified several fine-scale associations between CBCs, gene expression, and faecal microbiota composition. NAFLD is a common cause of chronic liver disease in Western countries and is linked to obesity, type 2 diabetes mellitus and cardiac pathologies. Here we show that the French DD herd is potentially affected by this syndrome.

## Introduction

Minipigs are a group of small-sized swine lines, which started to be developed in the 1940s for biomedical research. They present many advantages over conventional pigs, like size, ease of handling and decreased feed requirements^1^. Today, a large variety of breeds is available for very different applications. Minipig lines are maintained using specific breeding strategies, which minimize inbreeding while keeping the genetic integrity of the population^2^. Examples of minipigs include the MeLiM line, a model to study an inheritable form of melanoma undergoing spontaneous regression^3^ and the Yucatan strain, used for research on cardiovascular pathologies and diabetes^1^. Minipig lines homozygous at the major histocompatibility locus (MHC, also known as Swine Leukocyte Antigen or SLA in pigs) were also produced by the NIH, mostly for immunology studies and transplantation programs^4^.

Non-alcoholic fatty liver disease is the most common cause of chronic liver disease in developed countries^5,6^. It spans a spectrum of pathologies ranging from hepatic steatosis to non-alcoholic steatohepatitis (NASH) and cirrhosis^6–8^. While NAFLD was traditionally seen as a pathology affecting mainly the liver, it has recently been shown that its burden is not limited to this organ. There is increasing evidence that NAFLD is multisystemic, involving extra-hepatic organs and pathways. It is associated with obesity^9^, type 2 diabetes mellitus, insulin resistance, metabolic syndrome and cardiovascular diseases^5–7,10–12^. Despite the complexity of this pathology, many NAFLD-related molecular pathways are known^13–15^, and its development is usually modelled using the “two-hit hypothesis”^12,13^. The “first hit” corresponds to insulin resistance-mediated fat accumulation in hepatocytes. The “second hit” leads to hepatocyte injury, inflammation and fibrosis and is triggered by oxidative stress and proinflammatory cytokines^12^. Subsequently, cytokines interact with their receptors to initiate various signalling cascades^14–16^. Since NAFLD is linked to cardiac diseases, other processes such as platelet activation are involved^10^.

To date, rodents have been the pillar for research on obesity, NAFLD and NASH, but physiological differences with humans slow their use^15,17^. Alternative models are being pursued, and the pig is acknowledged as a relevant one because of its anatomical, physiological, and metabolic similarities with humans^15,17–19^. Minipigs show a distinct tendency towards obesity and related pathologies^20^, and several lines are used for their study, such as Ossabaw, Göttingen, and Bama^15,17,21^. In the aforementioned papers, the animals were fed specific high-calorie diets, but most minipigs develop obesity even when receiving *ad libitum* standard chow^1,20,22^. Indeed, the recommended feed intake for minipigs corresponds to 40% *ad libitum* intake^23^.

The inbred DD line was created and maintained homozygous at the SLA locus, harbouring the Hp-4.4 haplotype (IPD-MHC database, https://www.ebi.ac.uk/ipd/mhc/)^24^. DD pigs were brought to France more than 30 years ago and maintained inbred thereafter. They do not seem especially susceptible to obesity as the DD pigs imported to Great Britain (Mick Bailey, personal communication), but they have not been characterized for any metabolic disease yet.

Our aim was to study the physiological status of the French herd of DD pigs by comparison to commercial Large White (LW) pigs. We measured three sets of intermediate phenotypes^25^, i.e. complete blood counts (CBCs), whole blood transcriptome profiles and faecal DNA microbiota characterized by 16S rRNA gene sequencing. Integrative analysis suggested that the DD pigs were potentially affected by a disease of the NAFLD spectrum.

## Results

We studied 12 DD and 12 LW contemporary pigs, measuring CBCs, blood transcriptome and faecal microbiota at 60 days of age. CBCs were also measured at four other time-points to provide a time-course that was used to confirm the data obtained at 60 days. The total number of animals used for the analyses ranged from 17 to 24 according to the measures and the time-points (Supplementary Table 1).

### The LW and DD pigs differ by CBC parameters

CBCs were measured from blood sampled at 8, 20, 40, 60 and 100 days of age. Because some data were missing, the datasets comprised 23, 16, 24, 20 and 17 samples, respectively (Supplementary Table 1). Eighteen parameters were measured at each time-point (Supplementary Table 2, Supplementary Fig. 1).

Twelve parameters were differentially abundant between DD and LW pigs at least at one time-point. After quality control, five parameters were discarded as non-reliable. The other seven were split in two groups displaying opposite patterns (Supplementary Table 3A). The metrics of the first group showed higher values in DD pigs (Supplementary Table 3A). They were related to viscosity and platelet activation, and included hematocrit (hct), mean corpuscular volume (mcv), mean corpuscular hemoglobin (mch), hemoglobin (hgb), and platelet distribution width (pwd).

The hct and the derived metric mcv were significantly higher in DD pigs over the whole time-course of the experiment. In the case of mch, hgb and pwd, the minipigs also presented higher values during all the period, but some time-points lacked statistical support (Supplementary Table 3A).

The second group of differentially abundant CBC parameters was linked to inflammation and included metrics related to white blood cells. These parameters tended to be lower in DD pigs (Supplementary Table 3A). Monocyte absolute number (monum) and monocyte percentage (mopro) were decreased in DD pigs, but with some inconsistencies and low statistical support.

**Figure 1:**
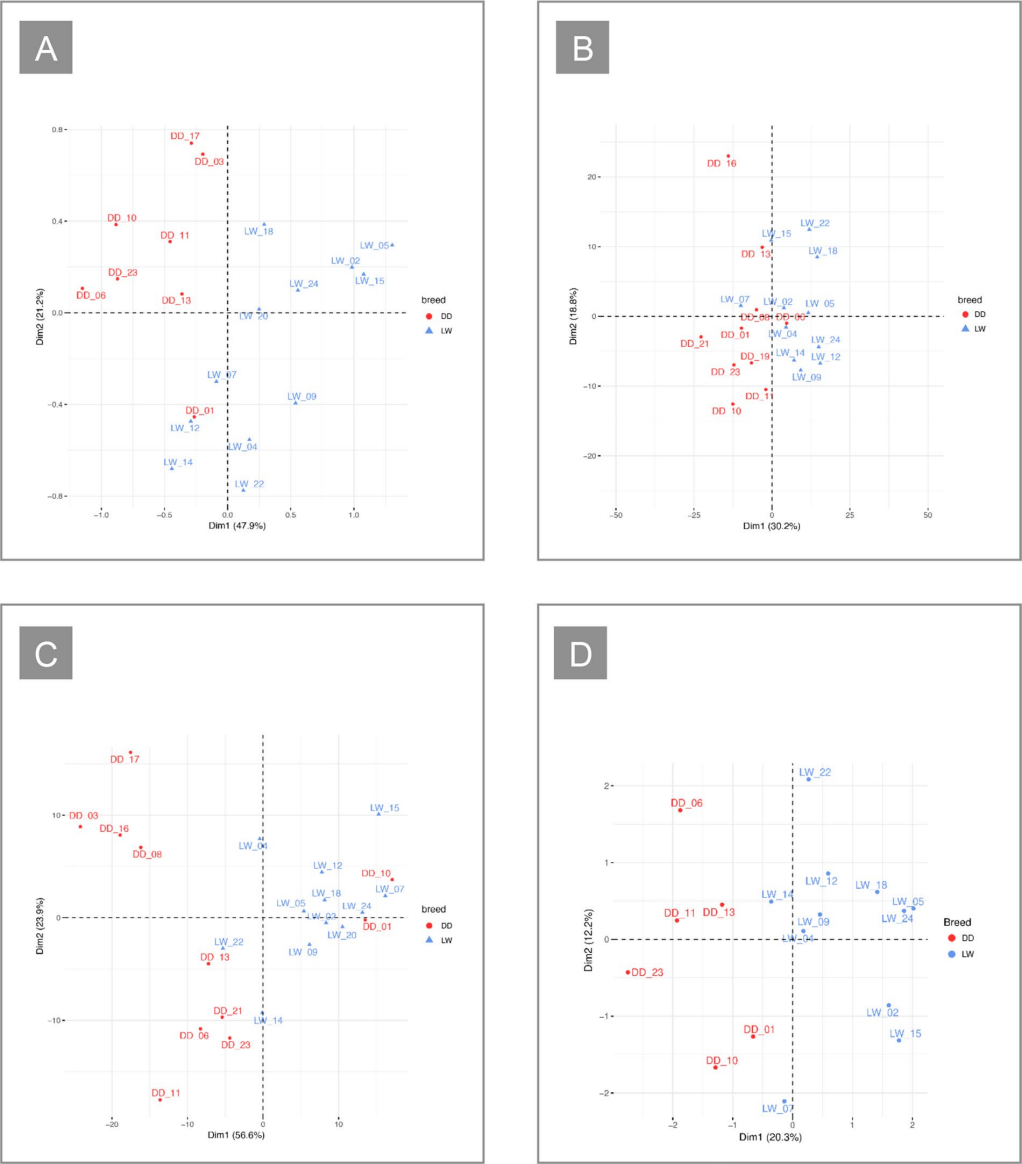
(**A**) Plot of the first two components of the PCA based on the CBC data. **(B)** Plot of the first two components of the PCA based on the expression values of all the genes expressed in the blood. **(C)** Plot of the first two components of the PCA based on the faecal microbiota data. **(D)** Plot of the first two components of the IFM obtained from the MFA analysis performed on CBCs, genes, and the microbiota data.

A principal component analysis (PCA) separated the two breeds, with the first component accounting for 47.9% of the total variability (Fig. 1A). Hierarchical clustering, instead, did not found a clear split (Supplementary Fig. 2A).

### Blood transcriptomes of DD and LW pigs revealed a potential NAFLD syndrome in DD pigs

Blood transcriptome profiles were obtained from 60-day-old animals. After quality assessment, 21 samples were retained (Supplementary Table 1). A total number of 3,046 differentially expressed (DE) genes were identified (Supplementary Table 4), of which 1,405 were upregulated and 1,641 downregulated in DD pigs. The values of log_2_ fold change (FC) ranged from 4.1 to −10.7, but were generally low. In fact, only 38 genes presented a log_2_ FC higher than 1.5 or lower than −1.5. The 20 most upregulated and downregulated genes in DD pigs compared to LW pigs are listed in Table 1.

**Table 1:** Table listing the 20 highest and the 20 lowest differentially expressed genes in whole blood in DD pigs respect to LW pigs. The second column shows gene descriptions, the third one the log_2_ FC values and the fourth one the Benjamini-Hochberg-adjusted p-values.

Gene	Gene description	log2(FC)	BH adjusted p-value
IFITM1	Interferon induced transmembrane protein 1	4.11	0.001878
REXO2	RNA exonuclease 2 homolog	3.96	0.000044
COL16A1	Collagen type 16 alpha 1 chain	2.87	0.000001
PCP4	Purkinje cell protein 4	2.58	0.000191
GSG1	Germ cell associated 1	2.57	0.000303
TOR3A	Torsin family 3 member A	2.45	0.000165
LGALS7	Lectin galactoside-binding soluble 7	2.29	0.000616
SPINK4	Serine peptidase inhibitor Kazal type 4	2.25	0.000001
LY6D	Lymphocyte antigen 6 family member D	2.15	0.000229
SYT9	Synaptotagmin 9	2.11	0.000314
HEATR4	HEAT repeat containing 4	2.11	0.000473
KAZALD1	Kazal-type serine peptidase inhibitor domain 1	2.07	0.000025
OAS1	2′-5′-oligoadenylate synthetase 1	1.87	0.013548
CXCL10	Chemokine (C-X-C motif) ligand 10	1.86	0.017611
BCL2L15	BCL2 like 15	1.76	0.001981
RSPH9	Radial spoke head 9 homolog	1.68	0.000935
CREB3L3	CAMP responsive element binding protein 3 like 3	1.56	0.000325
DNAH9	Dynein axonemal heavy chain 9	1.51	0.000094
SRPK3	SRSF protein kinase 3	1.50	0.000033
LOC106505804	Uncharacterized protein	1.48	0.000128
LOC100515340	Uncharacterized protein	−1.51	0.030272
VPREB1	V-set pre-B cell surrogate light chain 1	−1.52	0.000723
LOC100510923	Uncharacterized protein	−1.59	0.000180
HPGD	15-hydroxyprostaglandin dehydrogenase	−1.66	0.000153
RGS18	Regulator of G protein signaling 18	−1.68	0.000723
CRISPLD2	Cysteine rich secretory protein LCCL domain containing 2	−1.69	0.000002
F2R	Coagulation factor II thrombin receptor	−1.76	0.000036
ATOX1	Antioxidant 1 copper chaperone	−1.85	0.006059
FAM151B	Family with sequence similarity 151 member B	−1.89	0.000000
ZSCAN25	Zinc finger and SCAN domain containing 25	−1.91	0.000000
KCNQ3	Potassium voltage-gated channel subfamily Q member 3	−1.95	0.000005
OAZ3	Ornithine decarboxylase antizyme 3	−1.98	0.001265
MRPL42	Mitochondrial ribosomal protein L42	−2.09	0.000000
TAGLN	Transgelin	−2.11	0.008512
LAMB3	Laminin subunit beta 3	−2.15	0.000010
TMEM98	Transmembrane protein 98	−2.27	0.000263
TRDV3	T cell receptor delta variable 3	−2.32	0.000000
TCRA	T cell receptor alpha locus	−2.34	0.000000
ECHDC1	Ethylmalonyl-CoA decarboxylase 1	−3.46	0.000000
IFITM3	Interferon induced transmembrane protein 3	−10.69	0.000000

Several exploratory approaches were used to gather information about the structure of the dataset. A PCA was carried out using the expression values of all the genes. The first component accounted for 30.2% of the total variability and separated the two breeds (Fig. 1B). A clustering analysis confirmed this split, but three samples were misplaced (Supplementary Fig. 2B).

The functional analysis of the expression profiles was performed using several methods. First, the DE genes were processed using Ingenuity Pathway Analysis (IPA). Forty-six enriched pathways were found, 22 of which (or 47.8%) were related to NAFLD (Supplementary Table 5), like for example “adipogenesis”, “sirtuin signalling”, “mTOR signalling”, “PI3K/Akt signalling”, “B Cell receptor signalling”, and “hepatic fibrosis”^13,14,26,27^.

**Figure 2:**
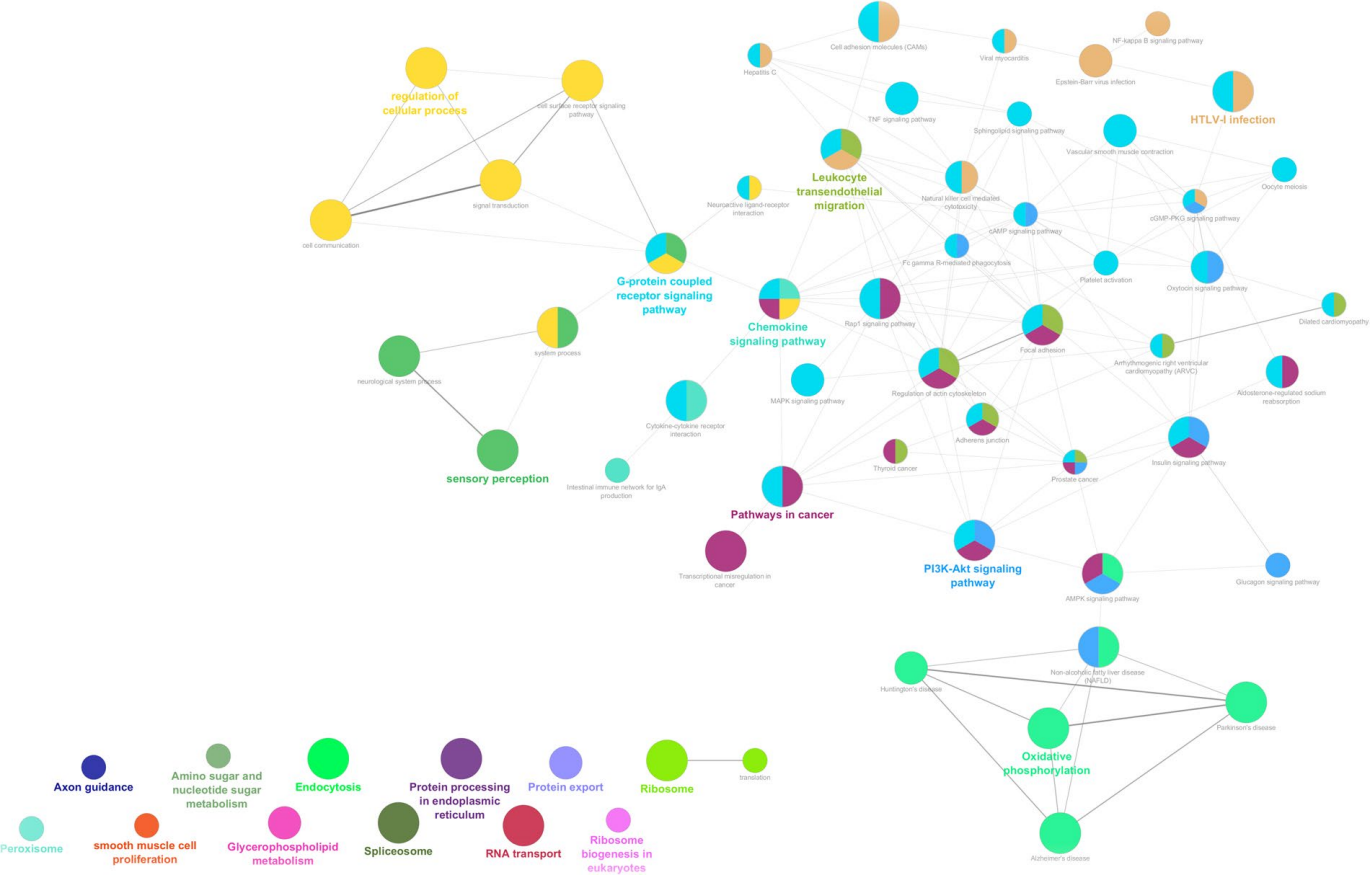
Representation of the functionally connected networks obtained using ClueGO 2.3.4^28^. Each node corresponds to an enriched GO term, and each colour corresponds to a GO group. The same term can be included in several groups, and its size reflects its statistical significance (refer to Supplementary Table 6). The 13 nodes at the bottom left of the picture are not connected to the network. Line width is proportional to k-score values.

A second analysis was performed with ClueGO 2.3.4^28^. Fifty connected terms were found, 48 of which presented a group adjusted p-value < 10^−10^ (Fig. 2), and 13 of which (or 27.1%) fitted into the NAFLD spectrum, like “non-alcoholic fatty liver disease”, “actin cystoskeleton”, “PI3K/AKT signalling”, “natural killer cell-mediated cytotoxicity”, and “leukocyte transendothelial migration” (Supplementary Table 6).

As a third step, a Gene Set Enrichment Analysis (GSEA)^29^ was carried out. Eleven MSigDB gene sets^30^ were enriched at a FDR q-value < 0.05, 4 of which (or 36.4%) were related to NAFLD (Supplementary Table 7). The results were globally consistent with those of IPA and ClueGO, as in the case of the signatures “insulin resistance (pancreas β cells)” and “mTOR signalling”.

The pathways related to NAFLD were evenly distributed across all the steps of the two-hit model and were often confirmed by two or more methods of analysis.

Processes such as those related to insulin resistance (ClueGO, GSEA) corresponded to the first hit, while pathways such as “oxidative phosphorylation” (IPA, ClueGO, GSEA) and “cytokine-cytokine receptor interaction” (ClueGO) represented the mechanisms leading to the second hit^12^. As found by others^13,14,26^, a significant proportion of processes (i.e. 43.5% on average) was related to signalling mechanisms, such as “mTOR signalling” (IPA, GSEA) and “ERK/MAPK signalling” (IPA, ClueGO).

The second hit was represented by pathways such as “hepatic fibrosis” (IPA) and “integrin signalling” (IPA), while the ontologies related to inflammation and to the immune response in obesity, like “leukocyte migration” (IPA, ClueGO), “phagocytosis” (IPA, ClueGO), and “cell adhesion” (IPA, ClueGO) were particularly abundant.

Because these three steps of analysis suggested the potential presence of an NAFLD-related pathology, we decided to explore the genes directly related to this disorder in more detail. For this, a literature-based meta-analytic approach was chosen. First, a non-redundant consensus list of 2,551 NAFLD-related genes was created (see methods). Among them, 1,679 were represented on the microarray and 386 were differentially expressed (Supplementary Table 4). The enrichment within the DE genes list was assessed using a Fisher’s exact test and a hypergeometric test, both of which were significant (respectively 0.0195 and 0.0104).

### DD and LW pigs differ by their faecal microbiota

The V3-V4 16S rRNA region was sequenced on 24 60-day-old animals. After quality checks, one low quality sample with less than 300 reads was discarded. Thus, 23 samples were retained for the analysis (Supplementary Table 1), with an average number of 2,180 reads per sample and a range of variation from 1,359 to 3,088 reads per sample. The sequences retained after pre-processing were used for the open reference-based operational taxonomic units (OTUs) picking using the Greengenes database (v. 13.8). A total of 1,224 OTUs and 49 genera were identified.

**Figure 3:**
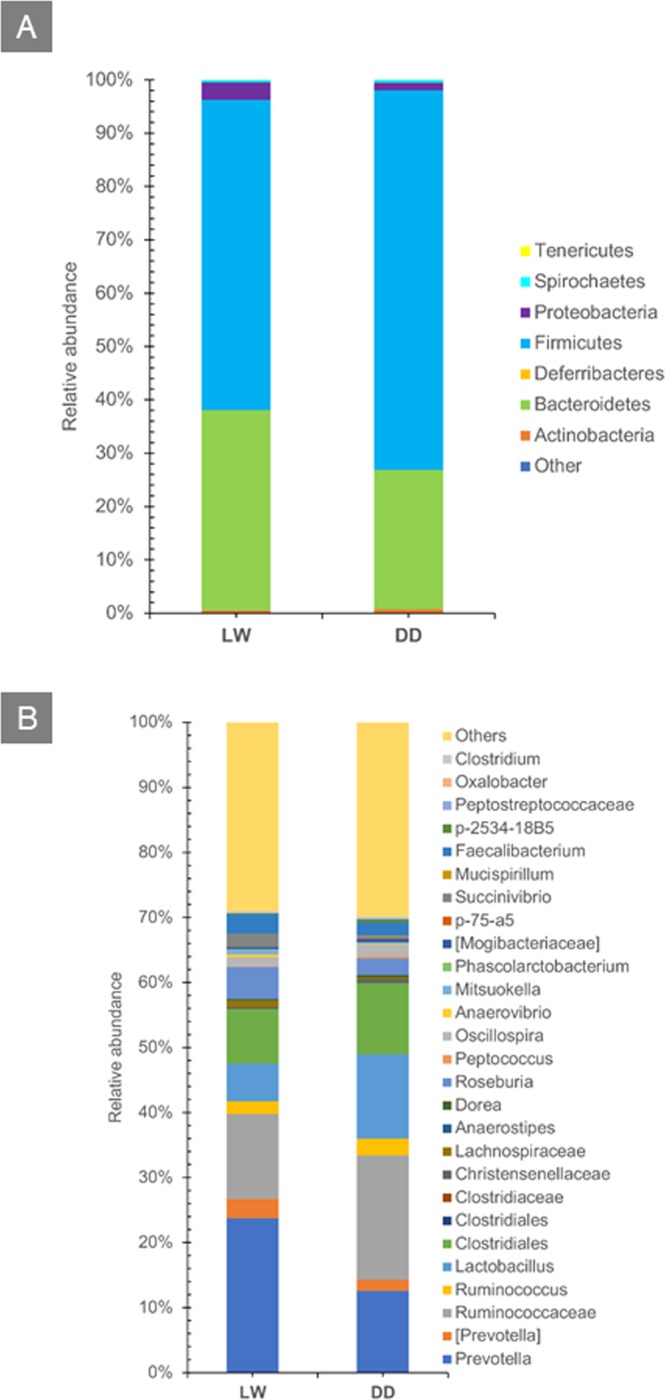
(**A**) Histogram representing the relative abundance (%) of phyla in DD (on the right) and LW (on the left) pigs. (**B**) Histogram representing the relative abundance (%) of microbial taxa in DD (on the right) and LW (on the left) pigs.

The values of α-diversity (OTU richness and Shannon diversity) did not differ between the two breeds, while β-diversity analyses suggested that taxonomic abundance profiles were significantly different between the groups, with p-values of 0.001 for both the PERMANOVA and the ANOSIM analyses (Supplementary Fig. 3). In agreement with the literature^22,31^, more than 95% of the sequences in both breeds were represented by the phyla Firmicutes and Bacteroidetes (Fig. 3A), followed by Proteobacteria, Actinobacteria and Spirochaetes. Firmicutes were significantly more abundant in DD minipigs, while Bacteroidetes and Proteobacteria were significantly less abundant (Supplementary Table 8). The two most abundant genera in both breeds were *Prevotella* and *Lactobacillus* (Fig. 3B). *Peptococcus*, *Lactobacillus* and *Ruminococcus* were significantly more abundant in DD pigs, while *Anaerovibrio*, *Prevotella*, *Succinivibrio*, *Mitsuokella* and *Roseburia* were significantly less abundant (Supplementary Table 9).

To further characterize the composition of DD pigs microbiota, we compared our results to three available datasets, the first one obtained on NASH-affected Ossabaw minipigs^32^ and the other two obtained on obese Göttingen and obese Ossabaw minipigs, respectively^22^.

In the first comparison (i.e. the contrast between DD and LW pigs *versus* the contrast between NASH-affected Ossabaw and lean Ossabaw pigs), 14 taxa were common to the two studies, seven of which were significantly differentially abundant in both cases, and three of which showed the same direction of variation (Supplementary Information 2). This was the case with Firmicutes, which were more abundant in both the contrasts, and with Bacteroidetes and *Roseburia*, which were less abundant. In the second comparison (i.e. the contrast between DD and LW pigs *versus* the contrast between obese Göttingen and lean Göttingen pigs), seven taxa were shared by both studies. Only Firmicutes and Bacteroidetes showed statistically different abundances in the two studies, but with opposite directions of variation. The other five taxa were not significantly different in our study, and only three of them showed the same direction of variation. In the third comparison (i.e. the contrast between DD and LW pigs *versus* the contrast between obese Ossabaw and lean Ossabaw pigs) we found eight taxa common to both studies, six of which with the same direction of variation, i.e. Actinobacteria, Prevotellaceae, *Clostridium*, *Streptococcus*, *Bacteroides* and *Prevotella*. Of note, none of these contrasts was however statistically supported (Supplementary Information 2).

Both the PCA and the clustering analyses separated the two breeds, but several samples were misplaced (Fig. 1C, Supplementary Fig. 2C).

### Integration of the different layers of phenotype information

First, a multiple factor analysis (MFA) was performed using CBCs, blood transcriptome, and faecal microbiota data. The individual factor map (IFM) revealed a split between the breeds, with the first component accounting for 20.3% of the total variability (Fig. 1D). The variable group plot (Supplementary Fig. 4) showed that CBCs gave the highest contribution to the first dimension of variability and the lowest contribution to the second dimension, while transcriptome data showed the opposite pattern. Microbiota data presented intermediate values.

**Figure 4:**
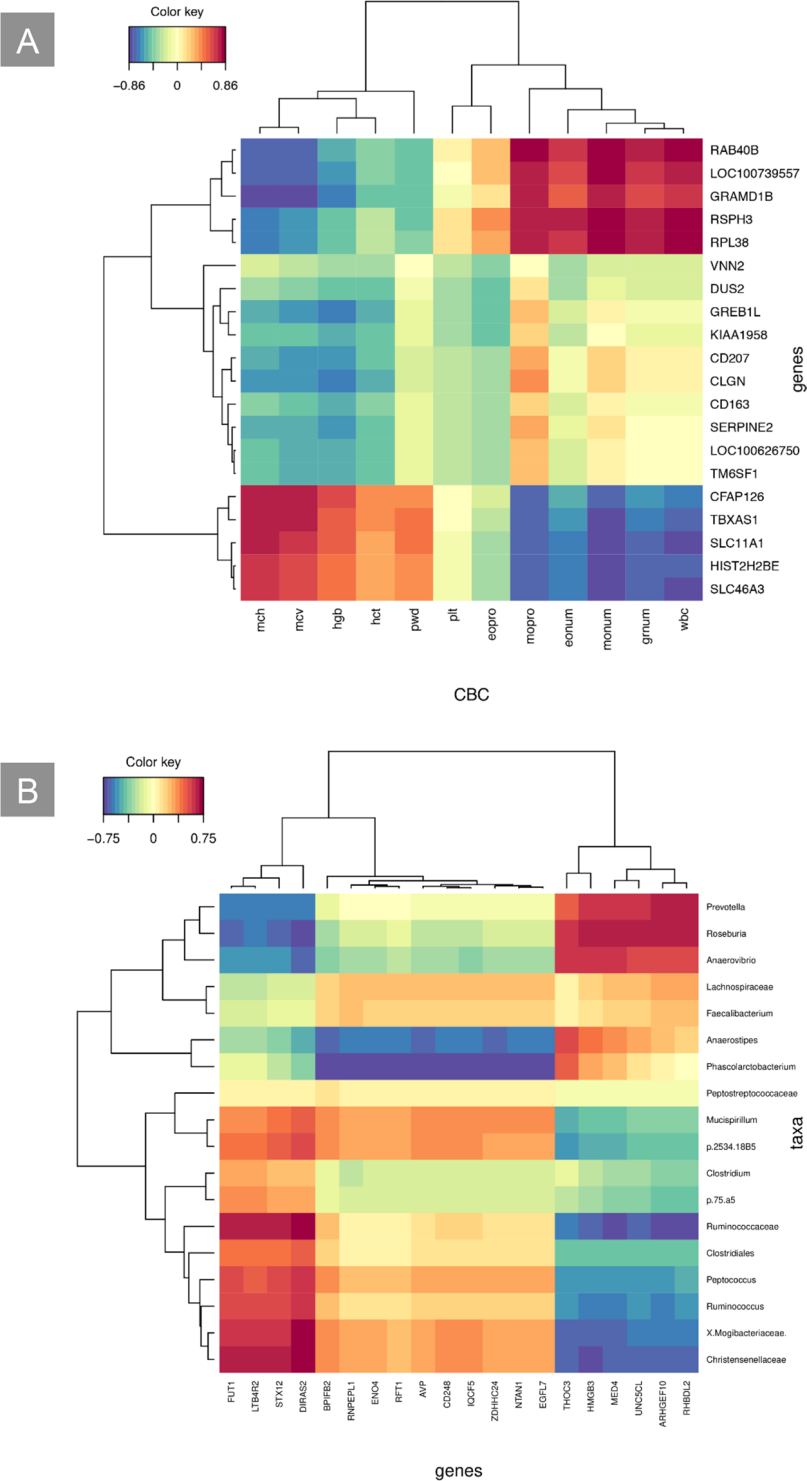
(**A**) A clustered image map (CIM) based on the association values obtained from the first two dimensions of the sPLS analysis integrating CBCs and DE genes. The CBCs parameters are plotted on the X-axis, and the genes are plotted on the Y-axis. The red and the blue coloured blocks indicate a positive or a negative association respectively, while the yellow colour indicates weak levels of association. **(B)** A clustered image map (CIM) based on the association values obtained from the first two dimensions of the sPLS analysis integrating DE genes in the blood and faecal microbial taxa. The DE genes are plotted on the X-axis, and the microbial taxa are plotted on the Y-axis. The red and the blue coloured blocks indicate a positive or a negative association respectively, while the yellow colour indicates weak levels of association.

Subsequently, the links between the different data layers were studied in a pairwise fashion with sPLS^33^ and using the data obtained at 60 days. In the case of CBCs and transcriptome, 17 individuals were used. The results were visualized producing a clustered image map (CIM)^34^, showing that CBCs were split in two main groups, corresponding to the metrics which had already been found more abundant and less abundant in DD pigs (Fig. 4A).

The most relevant associations were represented by 20 genes and 12 CBCs. Some CBC metrics, which were not differentially abundant between DD and LW pigs, did show association with DE genes (Supplementary Table 3B). Indeed, eonum, eopro, grnum and wbc grouped with the parameters showing lower abundance in minipigs. The metric eopro was discarded as not reliable (See methods).

In the case of transcriptome and faecal microbiota, sPLS was performed on 20 individuals. A CIM (Fig. 4B) revealed that the most important associations concerned 20 genes and 18 taxa. The genes were split into three groups. The first included four genes with strong positive association with Firmicutes such as *Peptococcus* and *Ruminococcus* and strong negative association with *Prevotella* and *Roseburia*, while the second included six genes showing the opposite pattern. The third group showed weaker association with the taxa.

For CBCs and faecal microbiota, the sPLS was based on 20 individuals, but no significant association between the variables was found.

## Discussion

The DD minipig herd maintained inbred in France does not seem especially prone to obesity, but to date there have been no attempts to characterize it in terms of metabolic diseases. Usually, these disorders are studied by feeding the animals with high-fat and high-sucrose diets. However, by a general point of view, minipigs can develop obesity even when they receive *ad libitum* standard chow^22^. This has been observed, for instance, in the DD minipig herd maintained in the United Kingdom (Mick Bailey, personal communication). In our study, the animals were fed *ad libitum* with classical pig diet and their physiological status was studied at 60 days by comparison to LW pigs. This latter commercial breed was chosen because of its lean meat percentage^20,35^. We combined the results from three data layers, i.e. CBCs, transcriptome profiles, and 16S rDNA sequencing, and our findings suggested that DD pigs were potentially affected by a disease belonging to the NAFLD spectrum.

First, we studied the overall data structure. The MFA showed a clear split between DD and LW pigs, which was globally confirmed by the PCA analyses individually performed on each dataset. In the case of hierarchical clustering, the split between the breeds was clearly supported by the transcriptome data alone. The transcriptome was also the most informative dataset in terms of contribution to the total variation. The microbiota contributed to a similar, but slightly lower extent, while the role of CBCs was more limited. These findings were in agreement with those obtained by Mach and co-workers^31^ and was possibly related to the number of variables of each dataset, which was highest in the case of transcriptome and lowest in the case of CBCs.

Then, we studied each single dataset independently to understand the molecular mechanisms underlying our biological systems, and we used pairwise sPLS analyses to better characterize their interactions. Two important hematorheological parameters, i.e. hematocrit and hemoglobin, were significantly higher in DD pigs over almost the whole time-course, making the finding more robust. High levels of hematocrit and hemoglobin are associated with obesity and metabolic syndrome^36,37^, respectively, and both metrics are related to insulin resistance, NAFLD and NASH in humans^38–40^. Mean corpuscular volume was also higher in DD pigs; however, even though this parameter is increased in some liver pathologies, it does not seem to be linked to NAFLD^41^. The values of platelet distribution width and mean platelet volume were also consistently higher in DD pigs, indicating platelet activation. This process is linked to obesity and NAFLD in both pigs and humans^23,42–44^. Platelets participate in the process of liver inflammation^45^ and their activation is specifically predictive of cardiovascular diseases^46^, which are strongly associated with NAFLD^5,10^. Because these processes are deeply intertwined, it is difficult to state whether augmented values of pwd and mpv point to a NAFLD-related disorder, a cardiovascular disease, or a combination of both. However, in a multisystemic perspective, they might suggest the presence of cardiac diseases associated to NAFLD as comorbidities. In contrast, monocyte absolute number and monocyte percentage showed a trend towards decreased values in minipigs. Low levels of monocytes have been correlated to septic processes in patients affected by chronic liver diseases, possibly representing a response to an extensive and acute inflammatory process^47^. In any case, the low levels of statistical support did not allow us to draw strong conclusions in this regard.

Transcriptome profiling also suggested a disorder belonging to the spectrum of NAFLD in DD pigs. This result was especially interesting because the enriched processes were found across each step of the two-hit model^12,13^ and because they were often confirmed by more than one analysis method. Furthermore, the pathways identified as enriched showed high levels of agreement with the literature available for humans^13,14,48^ and for Bama pigs^15^. These findings indicated a global shift in gene expression and suggested an impact of the disease on the physiological state of the minipigs. The pathways detected using traditional functional analysis corresponded to different molecular processes involved in NAFLD, but almost none of them represented an NAFLD-centered gene set. Indeed, a specific enrichment was obtained only with ClueGO, because both the IPA and the GSEA databases lacked appropriate ontologies. Since we wanted to study the potential presence of NAFLD in a more targeted way, we followed a meta-analytic approach based on the literature and then we obtained a significant enrichment of our DE genes list. This supported our characterization of the transcriptome in terms of NAFLD, with particular relevance because it was based on experimental expression studies (See methods). Moreover, looking at the 20 highest and the 20 lowest expressed genes, we found a strong overrepresentation of NAFLD-related genes, which added up to 25% (i.e. 5 out of 20 genes). Indeed, the same value calculated on the whole list was just 12.7% (i.e. 386 out of 3,042 genes). The genes belonging to the pathways used for the other methods of enrichment analysis, instead, accounted for only 5% of the total. This indicated that the deepest changes in the transcriptome affected genes that were directly involved in NAFLD. It was the case, for instance, with *IFITM1* (log_2_ FC = 4.1), *COL16A1* (log_2_ FC = 2.9), *TOR3A* (log_2_ FC = 2.4), *LY6D* (log_2_ FC = 2.2), *CXCL10* (log_2_ FC = 1.9), *F2R* (log_2_ FC = −1,8) and *TAGLN* (log_2_ FC = −2.1), which have key roles in many studies performed in pigs^15^, mice^6,49,50^ and humans^14,51^.

In agreement with CBC data, a specific enrichment of pathways related to cardiac disorders was also found, like in the cases of “heme metabolism” (GSEA), “platelet activation” and “cardiomyopathies” (ClueGO). This finding is meaningful in a multisystemic perspective, which stresses the link between NAFLD and cardiovascular diseases^5^.

The sPLS provided further hints about the connections between the first two data layers (i.e. CBCs and blood transcriptome). On the one hand, it confirmed the importance of the differentially abundant CBCs in the definition of the NAFLD phenotype, showing strong association with some DE genes. Interestingly, the large majority of genes (18 out of 20) was not known to be related to NAFLD and could therefore represent new candidates. This was the case, for instance, with *CFAP126* and *HIST2HBE*, which showed strong positive association with hematorheological parameters and strong negative association with monocytes, and which grouped with the well characterized *SLC11A1*^49^ and *TBXAS*^6^ genes. On the other hand, the sPLS also indicated that some non-differentially abundant CBC parameters were strongly associated with DE genes, even though their biological meaning was not easy to interpret. This happened, for instance, with the metric eonum. A loss of eosinophils was reported in the adipose tissue of patients affected by NASH^52^, but it is not straightforward to compare these results to ours. Also, NAFLD is usually associated with high wbc^53^ and mpv^44^ values, which contrasts with our findings.

The characterization of microbiota was less clear-cut. On the one hand, the comparison of our data to those obtained in NASH-affected Ossabaw pigs^32^ and in obese Göttingen pigs^22^ highlighted only limited similarities. On the other hand, the microbiota of our DD pigs shared some characteristics with that of the Ossabaw breed which, according to the authors, displayed the features associated with obesity^22^. However, the lack of statistical support did not allow us to draw robust conclusions on this point. The Firmicutes to Bacteroidetes ratio was higher in DD (2.7) than in Large White pigs (1.6), which was also consistent with the pattern observed in obese Ossabaw minipigs^22^ and could provide some support to the presence of obesity^32,54^. However, these pieces of evidence were not sufficient to make robust inferences about microbiota data.

The sPLS analysis provided more details about the relationship existing between microbiota and DE genes. The genus *Prevotella*, for instance, was negatively associated with obesity in Ossabaw minipigs^22^ and was similarly underrepresented in DD pigs. According to our results, the genus *Prevotella* exhibited negative association with the genes *FUT1*, *LTB4R2*, *STX12* and *DIRAS2*, which could be therefore related to this pathology. Except for *LTB4R2*, these genes have never been associated with obesity or NAFLD and could be therefore new candidates for these disorders. The genera *Roseburia* and *Anaerovibrio* grouped with *Prevotella*, pointing to a potential link to the NAFLD spectrum; however, no evidence for the involvement of these bacteria in obesity was found in Ossabaw pigs^22^.

Taken together, our results suggest that the DD minipigs could potentially present symptoms of a pathology belonging to the NAFLD spectrum. It is important to refer to NAFLD spectrum, and not to NAFLD *stricto sensu*, because of the inherent complexity of this disorder and because we could not produce additional physiological data to allow a specific diagnosis. In any case, NAFLD is increasingly being regarded as a multisystem disease^5^, which implies that it its constitutively linked to a large array of comorbidities.

Even if all the data layers supported our hypothesis, each one highlighted a slightly different combination of pathologies. In the case of CBCs, the results pointed to the presence of cardiac diseases and NAFLD, with a stronger representation of the former. In the case of transcriptome, NAFLD seemed to be the primary disorder, while cardiac diseases appeared as comorbidities. Microbiota data, instead, suggested the potential presence of obesity, but this evidence was not strong.

Transcriptome was the most informative data layer, and its integration with CBCs and microbiota produced consistent results. Therefore, we can speculate that our tentative characterization of the datasets in terms of NAFLD could potentially be the most accurate.

It must be considered that some limitations affect our results. Our data were obtained on young individuals, while NAFLD-related disorders are typically studied on 4- to 30-month-old animals. Therefore, many symptoms were likely not completely apparent. Moreover, transcriptome and microbiota data were only obtained at 60 days, while more time-points could provide a better characterization. Nevertheless, our work adds useful information to characterize NAFLD-related disorders in pigs, providing a robust workflow for the analysis of intermediate phenotypes.

## Materials and Methods

### Animals and diet

Twelve LW (i.e. 6 pairs of siblings) and twelve DD pigs (i.e. 6 pairs of siblings) of the same age were used (Supplementary Table 1). The suckling piglets had access to a pre-starter feed from day 3 (18% crude protein DM, 7.5% crude fat DM, 1.55% lysine DM) and were weaned at day 28. They were fed a starter diet from day 29 to day 40 (19% crude protein DM, 7% crude fat DM, 1.45% lysine DM) and were fed a grower diet *ad libitum* (16.5% crude protein DM, 2.4% crude fat DM, 1.17% lysine DM) from day 41 to day 150.

All the animals were raised and sampled at the INRA experimental farm of Nouzilly (France) in 2012 (farm agreement E 37-175-2). All the experiments were compliant to the French regulation for use of animals in research (articles R214-87 to R214-137 of the French rural code before the legal update enacted in 2013). No ethics approval was required for the collection of blood samples under the then current regulation.

### Blood and stool sampling

The blood samples for CBCs were collected at 8, 20, 40, 60 and 100 days of age using BD Vacutainer EDTA tubes (Becton Dickinson, Franklin Lakes, NJ, USA). The blood samples for RNA extraction were collected from 60-day-old animals using PAXgene Blood RNA tubes (Qiagen, Hilden, Germany). Faecal aliquots (200 mg) were collected from the rectum of the animals at 60 days of age, snap-frozen in liquid nitrogen and stored at −80 °C.

### Complete blood counts: data production and analysis

CBCs were obtained using a scil Vet abc counter (scil animal care company, Altorf, France). Eighteen parameters were measured for each time-point: white blood cell count (wbc); red blood cell count (rbc); platelet count (plt); hemoglobin (hgb); hematocrit (hct); mean corpuscular volume (mcv); mean corpuscular hemoglobin (mch); mean corpuscular hemoglobin concentration (mchc); platelet distribution width (pwd); mean platelet volume (mpv); lymphocytes absolute number (lynum); monocytes absolute number (monum); granulocytes absolute number (grnum); eosinophils absolute number (eonum); lymphocytes percentage (lypro); monocytes percentage (mopro); granulocytes percentage (grpro); eosinophils percentage (eopro).

A linear-mixed model was fitted to each parameter using the lme4 R package^55^. The breed was set as a fixed effect and the sow as a random effect. Data of each day were separately analysed.

The results obtained on 60-day-old individuals were used for the subsequent analyses, while the values obtained at the other time-points (i.e. 8, 20, 40 and 100 days) were used only as a means of validation. A metric was retained only if the direction of the change in the differential abundance at 60 days (i.e. positive or negative) was consistent with at least two out of five points.

The metrics retained were rescaled using the ‘rescale’ function of the scales R package (https://CRAN.R-project.org/package = scales) and analysed through a PCA and a hierarchical clustering using the FactoMineR R package and selecting the Pearson’s correlation coefficient and the Ward method^56^.

### RNA extraction and microarray processing

Total RNA from blood of 60-day-old individuals was isolated using the PAXgene Blood RNA Kit (Qiagen). RNA quality and integrity were determined using a NanoDrop 1000 (Thermo Scientific, Waltham, MA, USA) and a Bioanalyzer 2100 (Agilent Technologies, Santa Clara, CA, USA).

Transcriptional profiling was performed using an Agilent 8 × 60K pig custom microarray (Agilent Technologies, AMADID 037880). Twenty-four samples were processed (i.e. three chips). All steps were performed by the @BRIDGe facility (INRA Jouy-en-Josas, France, http://abridge.inra.fr/), as described previously^57^. Microarray data were submitted to GEO (accession number GSE111953).

### Microarray data analysis

The annotation of the pig array was updated using the Sscrofa 11.1 version of the pig genome (see Supplementary Information 1).

Probe intensities were background corrected using the “normexp” method, log_2_ scaled and quantile normalized using the Limma R package^58^. The probes of the lowest quartile were filtered out, controls were discarded and the probes corresponding to genes were summarized.

The obtained expression matrix (“E1”) was processed with the arrayQualityMetrics R package^59^ for quality assessment. Three outliers and a technical bias concerning the third chip were detected. After outlier removal, the raw data were pre-processed again to obtain the “E2” expression matrix.

The differential analysis was performed using the Limma R package. A linear model was fitted for each gene, setting the breed and the chip as fixed effects, and comparing DD to LW pigs. The sow was included as a random effect using the “duplicateCorrelation” function. The p-values were Benjamini-Hochsberg corrected setting a threshold of 0.05.

An alternative linear model was tested, including also monocyte count as a fixed effect, but the small sample size (i.e. 17 individuals) determined a sharp decrease in the statistical power of detection.

The E2 expression matrix was further modified. In fact, this matrix was not corrected for the chip-related bias, which was treated as an effect by the Limma linear model. To perform other downstream analyses, this bias was corrected using the Limma “removeBatchEffect” function. The “E3” corrected matrix was used to perform a PCA and a clustering with FactoMineR^56^.

### Functional analysis of blood transcriptome

Transcriptome functional analyses were performed using four approaches. First, the DE genes were analysed with Ingenuity Pathways Analysis 01.08 (September 2017 Release, IPA, www.ingenuity.com) to identify canonical pathways. Only those with a -log(p-value) > 1.75 and including at least 10 genes were considered.

The second analysis was realized using ClueGO 2.3.4^28^. A two-sided test was used to highlight enriched GO terms. Significance was set at an adjusted p-value of 0.05, the “GO fusion” option was used, the k-score was fixed at 0.2 and the global/local parameter at the sixth level. Three ontologies were selected: “Sus scrofa ontologies KEGG_06.07.2015”, “GO_ImmuneSystemProcess_03.07.2015_09h08” and “GO_BiologicalProcess_03.07.2015_09h08”.

A third analysis was performed using GSEA^29^ as implemented by the Broad Institute (http://software.broadinstitute.org/gsea/index.jsp). The E3 matrix was analysed using the “h.all.v6.1” MsigDB^29^, choosing the “gene_set” option for the permutations.

In all these three cases, the percentage of pathways putatively related to the NAFLD spectrum was estimated by performing a comparison with a pathway consensus list obtained using 19 papers describing transcriptional profiling^6,8,14–16,49,50,60–69^ or data meta-analysis^7,51^. A further review paper was also added^27^. More details are found in Supplementary Information 2.

The fourth approach involved a meta-analytical screening of the literature relative to NAFLD and other related diseases. The same already listed 19 papers were used, and the available DE gene lists were retrieved and merged to obtain a non-redundant set, which was intersected with our DE gene list. The enrichment was assessed using a Fisher’s exact test and a hypergeometric test.

### Production and analysis of 16S data

DNA was extracted from frozen faecal samples as described by^70^. Subsequently, the V3–V4 region of the 16S rRNA gene was sequenced using the 454 GS FLX Titanium platform (Roche, Pleasanton, CA, USA) as described by^71^. FastQC was used to perform quality checks on the raw data and the sequences were analysed with QIIME 1.9.1^72^ as described previously^71^.

Species richness estimates (Chao1, observed OTUs, and abundance-based coverage estimators or ACE) and evenness indexes (Shannon and Simpson), were calculated using the phyloseq R package after the reads were rarefied^73^. For β-diversity, weighted UniFrac distances were first calculated on the OTUs table. Then, nonmetric multidimensional scaling (nMDS) and distances to centroid were calculated using the “betadisper” function of the vegan R package. Breed comparisons were assessed with PERMANOVA using the “adonis” function in the vegan R package^74^, and p-values were obtained using Monte Carlo random draws. Also, an ANOSIM was performed using the vegan R package to test the significance of the difference between the breeds.

A linear-mixed model was fitted on the OTUs table using the lme4 R package and setting the breed as a fixed effect and the sow as a random effect. Relative abundance (%) differences at phylum and lower taxonomic levels between DD and LW pigs were assessed using MetagenomeSeq 1.20.1^75^. A Kruskal-Wallis test was performed, and q-values were Benjamini-Hochberg adjusted. The differentially abundant taxa were compared to the results published in two recent papers^22,32^ by following the procedure described in Supplementary Information 3. A PCA and a clustering were also performed. 16S data were submitted to SRA (accession number SRP144235).

### Data integration

An MFA^76^ was used to integrate CBCs, blood transcriptome, and 16S data using the FactoMineR R package^56^. The results were visualized using an individual factor map (IFM) and a variable groups plot.

The datasets were then studied in a pairwise fashion with sPLS^33^ using the mixOmics R package (https://cran.r-project.org/web/packages/mixOmics/)^77^. For each dataset pair, the analysis was performed using only the 60-day-old individuals for which the data belonging to both datasets were available. For CBCs and transcriptome and for transcriptome and 16S, the statistical models were optimized using the “perf” function and setting the “ncomp” parameter to two, and a CIM was used to visualize the data. No valid model was obtained for CBCs and 16S data.

## Supplementary information

Supplementary information.

Supplementary information2.

Supplementary information3.

Supplementary information4.

Supplementary information5.

Supplementary information6.

Supplementary information7.

Supplementary information8.

Supplementary information9.

Supplementary information10.

Supplementary information11.

Supplementary information12.

Supplementary information13.

Supplementary information14.

Supplementary information15.

Supplementary information16.
