# Supplementary figures and images for: Integrative analysis of blood and gut microbiota data suggests a non-alcoholic fatty liver disease (NAFLD)-related disorder in French SLAdd minipigs
Source: Sci Rep. 2020 Jan 14;10:234. doi: 10.1038/s41598-019-57127-x (PMC6959234; doi:10.1038/s41598-019-57127-x)

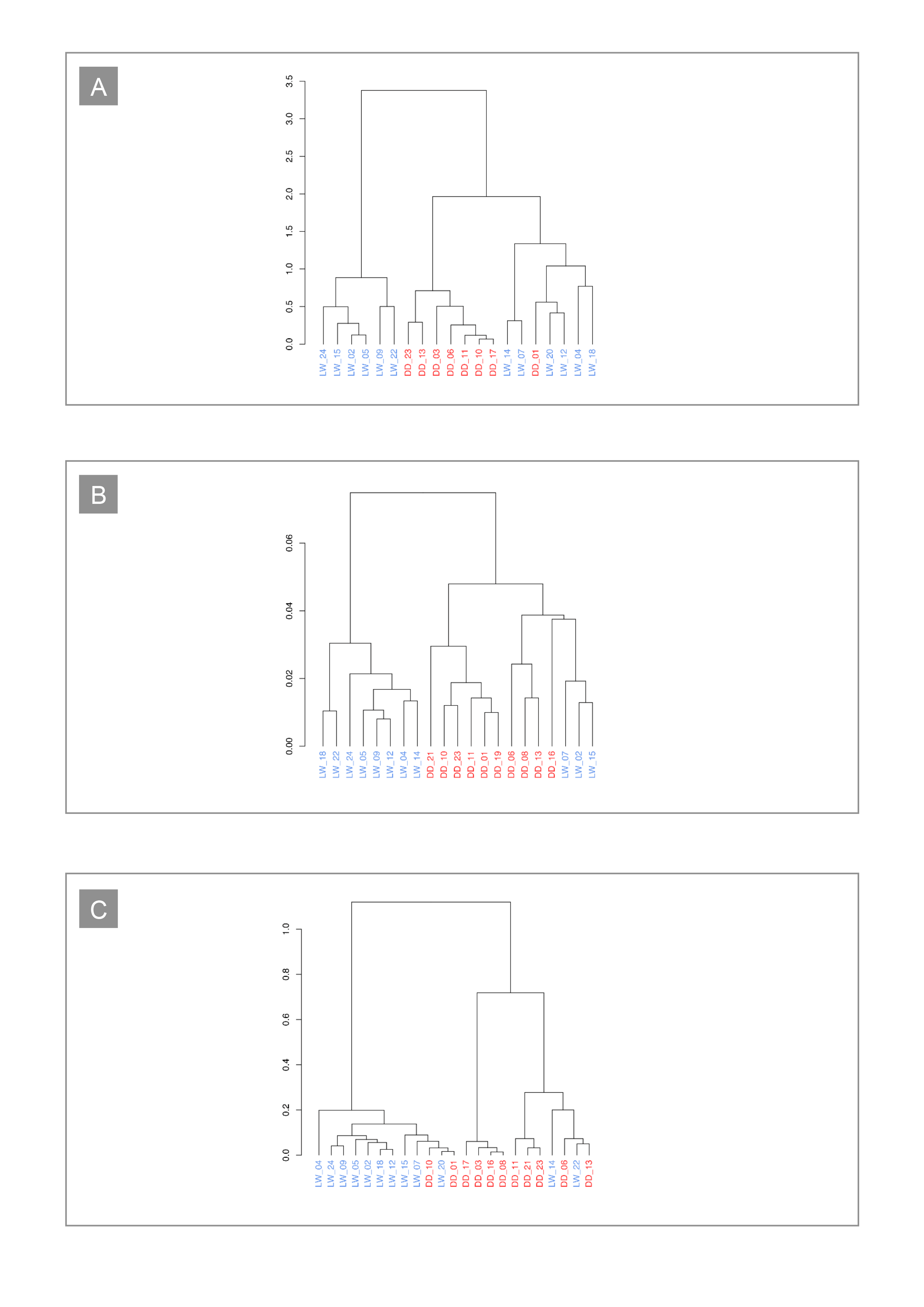

Supplement: Supplementary file 2 — Supplementary information2. [file 41598_2019_57127_MOESM2_ESM.tif]

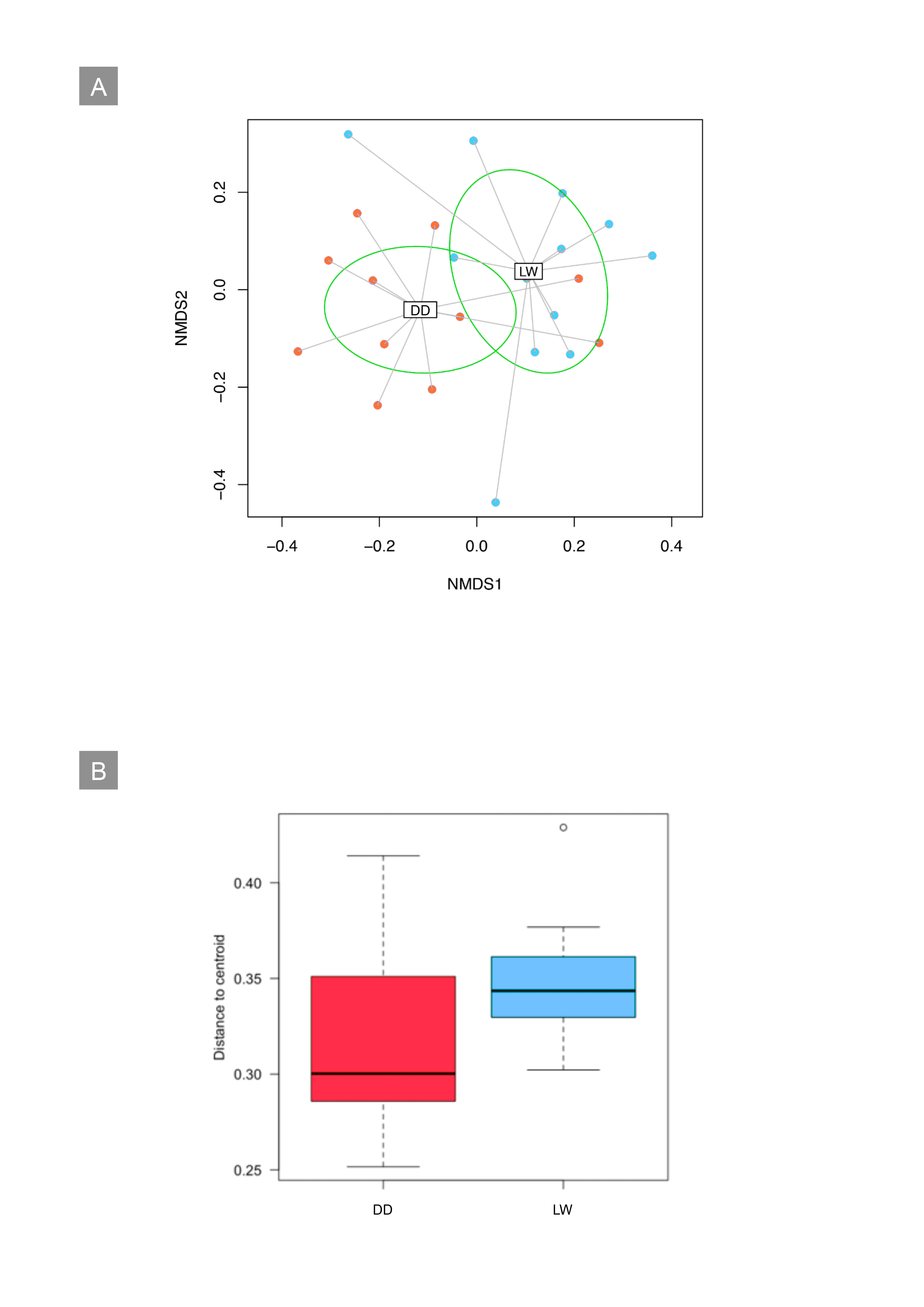

Supplement: Supplementary file 3 — Supplementary information3. [file 41598_2019_57127_MOESM3_ESM.tif]

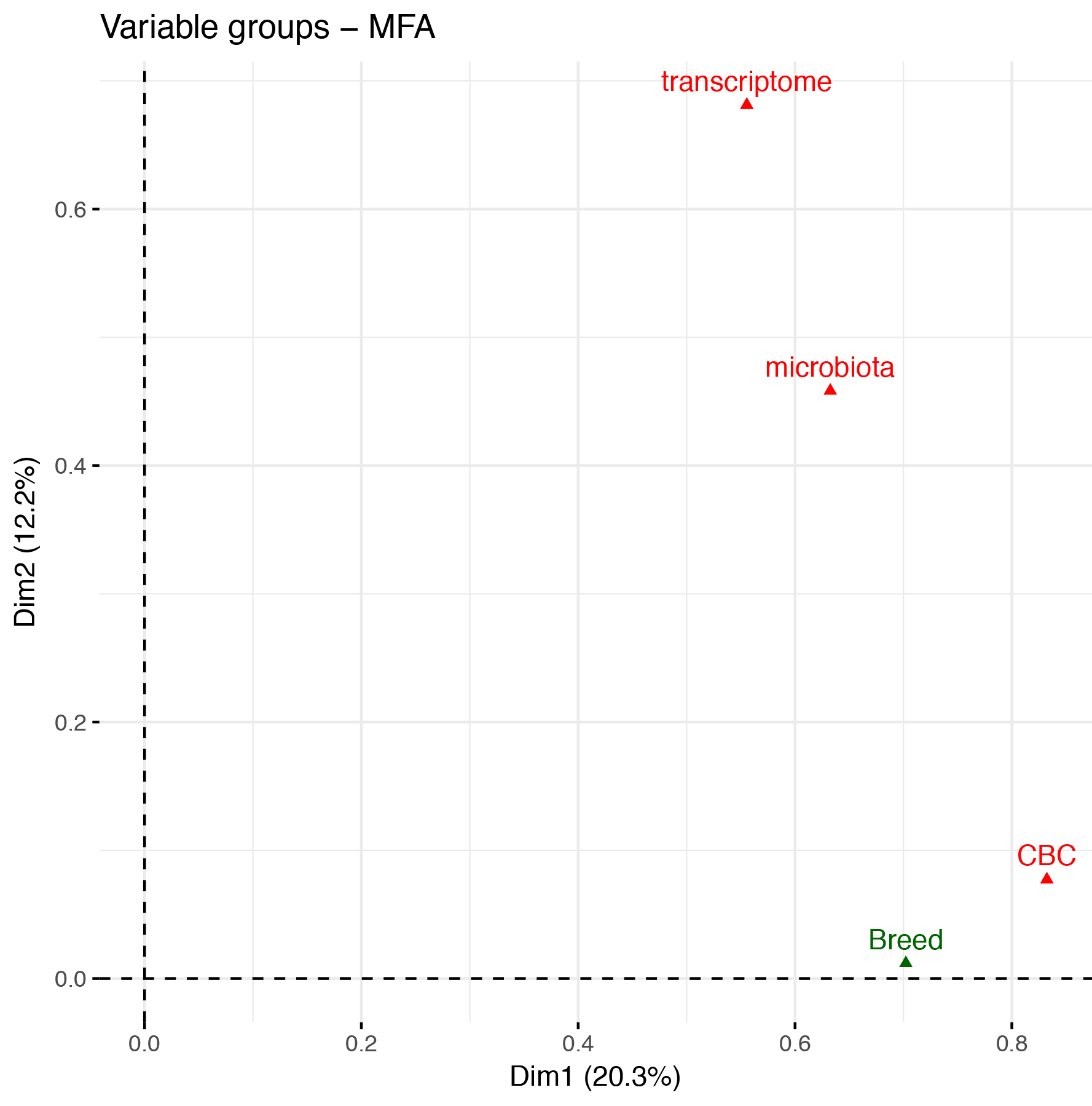

Supplement: Supplementary file 4 — Supplementary information4. [file 41598_2019_57127_MOESM4_ESM.tif]
