# Supplementary material for: Integrative analysis of blood and gut microbiota data suggests a non-alcoholic fatty liver disease (NAFLD)-related disorder in French SLAdd minipigs
Source: Sci Rep. 2020 Jan 14;10:234. doi: 10.1038/s41598-019-57127-x (PMC6959234; doi:10.1038/s41598-019-57127-x)
Supplement: Supplementary file 5 — Supplementary information5. [file 41598_2019_57127_MOESM5_ESM.docx]

**Supplementary information 1**. Detailed description of the workflow of annotation of the custom Agilent microarray.

---

**(I) Overview**

By a general point of view, the workflow of annotation of the pig Agilent chip was divided into five main steps:

1. the probes were mapped to the pig genome using TopHat and were then intersected to the proper annotation file. The details concerning the workflow used to perform this step are given in section (I)
2. the annotated probes were subsequently divided in four groups:
   1. probes which received an annotation both from NCBI and from Ensembl, and whose annotations were consistent. This group included 29,510 oligonucleotides
   2. probes which received an annotation only from NCBI or only from Ensembl. These two subgroups included 5,407 and 2,109 oligonucleotides, respectively
   3. probes which received an annotation both from NCBI and from Ensembl, and whose annotations were not consistent. This group was made up by 4,562 oligonucleotides
   4. probes which received an annotation from NCBI or Ensembl, and whose annotations corresponded to multiple different genes. This set corresponded to 2,076 unique oligonucleotides
3. the probes belonging to the groups (a) and (b) did not undergo further analysis
4. the probes belonging to the groups (c) and (d) underwent further analysis in order to solve the ambiguities found in their annotations. The details concerning the workflows used for the disambiguation of these probes are given in sections (III) and (IV)
5. the probe sets (a) and (b) where then merged to the disambiguated probe sets (c) and (d), and some minor steps of manual curation were performed. The final annotation included 40,730 probes, corresponding to 14,695 unique genes

**(II) Mapping of the probes to the genome**

1. an initial dataset made up of 60,306 probes was mapped to the pig reference genome available at NCBI (<ftp://ftp.ncbi.nlm.nih.gov/genomes/Sus_scrofa/>). The software TopHat v2.0.14 was used setting the option “max-multihits” to 5 and the option “library-type” to fr-unstranded, and choosing the “GCF_000003025.6_Sscrofa11.1_genomic.fna” file as a reference. A total number of 52,699 probes (87.3%) was mapped following this protocol. Subsequently, the Bedtools command “intersectBed” was run in order to find the overlap between the “accepted_hits.bam” file obtained as an output of the TopHat analysis and the “ref_Sscrofa11.1_top_level.gff3” pig annotation file. The final intersection file was then edited in order to obtain a .txt annotation file
2. the same initial probe set was mapped to the pig reference genome available at Ensembl (<ftp://ftp.ensembl.org/pub/release-90/gff3/sus_scrofa/>). The software TopHat v2.0.14 was used setting the option “max-multihits” to 5 and the option “library-type” to fr-unstranded, and choosing the “Sus_scrofa.Sscrofa11.1.dna.toplevel.fa” file as a reference. A total number of 52,497 probes (87.1%) was mapped following this protocol. Subsequently, the Bedtools command “intersectBed” was run in order to find the overlap between the “accepted_hits.bam” file obtained as an output of the TopHat analysis and the “Sus_scrofa.Sscrofa11.1.90.gtf” pig annotation file. The final intersection file was then edited in order to obtain a .txt annotation file

**(III) Workflow for the disambiguation of the probes belonging to the group (c)**

1. the 4,562 probes belonging to this group were analyzed by performing a manual blastn analysis against the whole NCBI dataset
2. if the results were consistent with the previous NCBI annotation and included also hits from species other than pig, then the Ensembl annotation was checked
3. if the gene was not found in the Ensembl annotation, then the NCBI annotation was kept
4. if the gene was found in the Ensembl annotation, but the results were not consistent between NCBI and Ensembl, the Ensembl annotation was further inspected by directly examining the genome browser
5. in fact, in some cases the locus found in the Ensembl annotation overlapped to several other genes. If the gene annotated by NCBI was found among all of the annotated genes, then the NCBI annotation was retained
6. if the NCBI annotation corresponded to a sequence marked as “LOC”, but the blastn results also include hits corresponding to the Ensembl annotation, then the Ensembl annotation was retained
7. if the NCBI annotation corresponded to a subject marked as “LOC”, but the Ensembl results did not show any precise name for the gene, a further blastn analysis on the probe was carried out as a potential confirmation
8. after these steps of annotation, a total number of 3,710 probes was retained, corresponding to 81.3% of the initial set

**(IV) Workflow for the disambiguation of the probes belonging to the group (d)**

1. the 2,076 probes belonging to this group were analyzed by performing an automated blastn analysis against the whole NCBI dataset
2. only the subjects showing 100% identity were kept
3. the subjects were subsequently manually inspected
4. when all the subjects corresponded to the same gene, the corresponding gene name was kept
5. when some subjects corresponded to a certain gene, while the other gave “NA” as a result, then the gene name was kept if it was found in at least 75% of the subjects
6. when the subjects corresponded to two or more different genes, the probe was discarded, except in a few cases in which a manual inspection was performed
7. the cases in which a probe received only “NA” subjects as a result were discarded as well
8. after these steps of annotation, a total number of 1,035 probes were retained, corresponding to 49.8% of the initial dataset
